# A step in the Delaunay mosaic of order *k*

**DOI:** 10.1007/s00022-021-00577-4

**Published:** 2021-03-16

**Authors:** Herbert Edelsbrunner, Anton Nikitenko, Georg Osang

**Affiliations:** IST Austria (Institute of Science and Technology Austria), Am Campus 1, 3400 Klosterneuburg, Austria

**Keywords:** Discrete Morse theory, Order-*k* Voronoi tessellations, Hyperplane arrangements, Order-*k* Delaunay mosaics, Rhomboid tilings, Weighted points, Power distance

## Abstract

Given a locally finite set $X \subseteq {{\mathbb {R}}}^d$ and an integer $k \ge 0$, we consider the function ${\mathbf{w}_{k}^{}} :{\mathrm{Del}_{k}{({X})}} \rightarrow {{\mathbb {R}}}$ on the dual of the order-*k* Voronoi tessellation, whose sublevel sets generalize the notion of alpha shapes from order-1 to order-*k* (Edelsbrunner et al. in IEEE Trans Inf Theory IT-29:551–559, 1983; Krasnoshchekov and Polishchuk in Inf Process Lett 114:76–83, 2014). While this function is not necessarily generalized discrete Morse, in the sense of Forman (Adv Math 134:90–145, 1998) and Freij (Discrete Math 309:3821–3829, 2009), we prove that it satisfies similar properties so that its increments can be meaningfully classified into critical and non-critical steps. This result extends to the case of weighted points and sheds light on *k*-fold covers with balls in Euclidean space.

## Introduction

Given a locally finite set $X \subseteq {{\mathbb {R}}}^d$ and non-negative integer *k*, the *order-k* *Voronoi tessellation* decomposes ${{\mathbb {R}}}^d$ into closed convex domains in which the *k* nearest points in *X* are the same [9, 14]. In other words, knowing the domain that contains a point $x \in {{\mathbb {R}}}^d$ is equivalent to knowing which *k* points of *X* are nearest to *x*. This motivates the use of the tessellation as a data structure for the *k*-nearest neighbor problem [3]. Similar to the ordinary (order-1) Voronoi tessellation, the order-*k* Voronoi tessellation has a natural dual [1], which we refer to as the *order-k* *Delaunay mosaic*, denoted ${\mathrm{Del}_{k}{({X})}}$. Its cells represent collections of domains with non-empty common intersection. We are interested in the function ${\mathbf{w}_{k}^{}} :{\mathrm{Del}_{k}{({X})}} \rightarrow {{\mathbb {R}}}$ that maps each cell to the minimum radius, *r*, such that the corresponding intersection of domains contains a point at distance at most *r* from each one of its *k* nearest neighbors. The sublevel sets of ${\mathbf{w}_{k}^{}}$ generalize the notion of alpha shapes from $k = 1$ to orders $k \ge 1$ [5, 13]. Recently, the stochastic properties of ${\mathbf{w}_{k}^{}}$ have been studied [6] and algorithms for computing the persistence have been presented [7]. We shed additional light on these results by establishing that ${\mathbf{w}_{k}^{}}$ behaves similar to a discrete Morse function. We hasten to mention that ${\mathbf{w}_{k}^{}}$ neither satisfies the requirements of a *discrete Morse function* [10] nor the slightly weaker requirements of a *generalized discrete Morse function* [11]. Nevertheless, we can classify the increments in the sublevel sets into critical and non-critical steps with predictable impact on the homotopy type. To state the result, we note that each cell of the order-*k* Delaunay mosaic is spanned by several size-*k* subsets of *X*, and we shall determine how to characterize incremental steps of ${\mathbf{w}_{k}^{}}$ in terms of such subsets. Specifically, taking any set of $\ell +1 \le d+1$ points in *X*, there is the unique smallest sphere that passes through these points. Assuming general position, the convex hull of these points is an $\ell$-simplex. Some of the size-*k* subsets of the $\ell +1$ points defining the sphere together with the points of *X* inside the sphere form cells of the Delaunay mosaic, and some of these cells constitute the unique step of ${\mathbf{w}_{k}^{}}$ corresponding to this sphere; see Sect. 2. Our main result is the following classification of the topology types of the steps:

- We call the configuration that defines a step *self-centered* if the center of the corresponding sphere is contained in the simplex spanned by the points on the sphere. Adding the cells in the step changes the Euler characteristic of the sublevel set, which implies that it also changes the homotopy type, so we refer to it as a *critical step* of ${\mathbf{w}_{k}^{}}$.
- We call the configuration *altruistic* if the center of the corresponding sphere is not contained in the simplex spanned by the points on the sphere. Adding the cells in the step preserves the homotopy type, so we refer to it as a *non-critical step* of ${\mathbf{w}_{k}^{}}$.

With minor adjustments, this classification extends to the case in which the points have real weights and the squared Euclidean distance is replaced by the power distance. This is most transparent when we view the Voronoi tessellations in ${{\mathbb {R}}}^d$ as projections of the levels in a hyperplane arrangement in ${{\mathbb {R}}}^{d+1}$; see e.g. [8]. Correspondingly, we view the Delaunay mosaics as horizontal slices of a rhomboid tiling in ${{\mathbb {R}}}^{d+1}$; see [7] for the construction in the unweighted case, which readily extends to points with weights.

*Outline* Section 2 introduces background on weighted Voronoi tessellations and hyperplane arrangements, their dual Delaunay mosaics and rhomboid tilings, and discrete Morse functions. Section 3 gives the proof of the main result, thus extending the framework of discrete Morse theory to include squared radius functions on order-*k* Delaunay mosaics. Section 4 concludes the paper.

## Background

It will be useful to see Voronoi tessellations as levels in hyperplane arrangements and Delaunay mosaics as slices of rhomboid tilings. We note that the different terminology for the *tilings*, *mosaics* and *tessellations* has roots in the different origins of the established terms, and we take advantage of it to simplify the distinction between the studied objects. In general, all these three notions refer to cell complexes whose cells are (possibly unbounded) convex polyhedra. To avoid redundancies, we explain everything for the more general, weighted case.

*Hyperplane arrangements* Let *X* be a locally finite set of points with real weights in ${{\mathbb {R}}}^d$, and let *J* be the corresponding *index set*. The *power distance* of a point $x \in {{\mathbb {R}}}^d$ from a weighted point $(x_j, w_j) \in X \subseteq {{\mathbb {R}}}^d \times {{\mathbb {R}}}$ is ${\pi _{j}{({x})}} = {\Vert {x}-{x_j}\Vert }^2 - w_j$. For $I \subseteq J$, the corresponding *(weighted) Voronoi domain* is the set of points that satisfy ${\pi _{i}{({x})}} \le {\pi _{j}{({x})}}$ for all $i \in I$ and $j \in J \setminus I$. For each non-negative integer *k*, the *(weighted) order-k* *Voronoi tessellation*, denoted ${\mathrm{Vor}_{k}{({X})}}$, is the collection of Voronoi domains for sets *I* that satisfy ${|{I}|} = k$. Setting $w_j = 0$ for all indices *j*, we get the unweighted situation as a special case.

To illuminate the structure of the order-*k* Voronoi tessellation, let $\varpi , f_j :{{\mathbb {R}}}^d \rightarrow {{\mathbb {R}}}$ be defined by mapping $x \in {{\mathbb {R}}}^d$ to $\varpi (x) = \tfrac{1}{2} {\Vert {x}\Vert }^2$ and to $f_j (x) = {\langle x , x_j \rangle } - \tfrac{1}{2} [{\Vert {x_j}\Vert }^2 - w_j]$. The graph of $\varpi$ is a paraboloid in ${{\mathbb {R}}}^{d+1}$, and the graph of $f_j$ is the hyperplane that touches the shifted paraboloid defined by $\varpi + \tfrac{1}{2} w_j$ in the point $(x_j , \varpi (x_j) + \tfrac{1}{2} w_j)$. We refer to the collection of hyperplanes as the *arrangement* of *X*. Let now $S = S(x, w)$ be the $(d-1)$-dimensional *sphere* with center $x \in {{\mathbb {R}}}^d$ and squared radius $w \in {{\mathbb {R}}}$. For $w > 0$ this is an ordinary sphere, for $w = 0$ it is a point, and for $w < 0$ it is what we call an *imaginary sphere*. There are different ways of visualizing the latter concept, but singularly important in this paper is that its squared radius is negative, giving the correct power distance if plugged into the formula given above. In either case, *S* partitions the index set into three subsets:

1 $$\begin{aligned} {\mathrm{In}{({S})}}&= \{ j \in J \mid {\pi _{j}{({x})}} < w \} , \end{aligned}$$

2 $$\begin{aligned} {\mathrm{On}{({S})}}&= \{ j \in J \mid {\pi _{j}{({x})}} = w \} , \end{aligned}$$

3 $$\begin{aligned} {\mathrm{Out}{({S})}}&= \{ j \in J \mid {\pi _{j}{({x})}} > w \} . \end{aligned}$$

In the unweighted case, when $w_j = 0$ for all indices *j*, the three sets correspond to the points inside, on, and outside the sphere. In the weighted case, the condition in (2) can be rewritten as ${\Vert {x}-{x_j}\Vert }^2 = w + w_j$, which we geometrically interpret as having two spheres, *S*(*x*, *w*) and $S(x_j, w_j)$, that intersect at a right angle. It is easy to see that $i \in {\mathrm{In}{({S})}}, {\mathrm{On}{({S})}}, {\mathrm{Out}{({S})}}$ iff $f_j(x) - \tfrac{1}{2} [{\Vert {x}\Vert }^2 - w] = \tfrac{1}{2} [w_j + w - {\Vert {x}-{x_j}\Vert }^2]$ is positive, zero, negative or, equivalently, iff the point $y(x) = (x, \tfrac{1}{2} [{\Vert {x}\Vert }^2 - w])$ in ${{\mathbb {R}}}^{d+1}$ lies below, on, above the graph of $f_j$. This motivates us to consider the decomposition of ${{\mathbb {R}}}^{d+1}$ defined by the hyperplanes. For each partition of *J* into three sets, we consider the corresponding partition of the set of the hyperplanes in ${{\mathbb {R}}}^{d+1}$, and define the corresponding *cell* to consist of points that at the same time are *on or under* the hyperplanes in the first set, *on* the hyperplanes in the second set, and *on or above* the hyperplanes in the third set. Important for us is the case when the three-partition is defined by a fixed sphere *S*. Then the corresponding cell consists of points $(x,z) \in {{\mathbb {R}}}^{d+1}$ that satisfy $z \le f_j(x)$ if $j \in {\mathrm{In}{({S})}}$, $z = f_j(x)$ if $j \in {\mathrm{On}{({S})}}$, and $z \ge f_j(x)$ if $j \in {\mathrm{Out}{({S})}}$. Assuming general position, this cell has dimension $d-p = d+1-{|{{\mathrm{On}{({S})}}}|}$. We refer to the $(d-p)$-dimensional cells as $(d-p)$*-cells* and to the $(d+1)$-cells as *chambers*. We write ${\mathrm{Arr}{({X})}}$ for the set of all cells. The sphere defines a chamber if ${\mathrm{On}{({S})}} = \emptyset$, and in this case we call $k = {|{{\mathrm{In}{({S})}}}|}$ the *depth* of the chamber, because there are precisely *k* hyperplanes above it. The relation between the chambers and the Voronoi domains should be clear.

### Proposition 2.1

(Chambers and Domains [8]) Let *X* be a locally finite set of points with real weights in ${{\mathbb {R}}}^d$. For every non-negative integer *k*, there is a bijection between the domains of ${\mathrm{Vor}_{k}{({X})}}$ and the chambers at depth *k* of ${\mathrm{Arr}{({X})}}$ such that every Voronoi domain is the vertical projection of its corresponding chamber to ${{\mathbb {R}}}^d$. $\square$

*Rhomboid tiling* To dualize the Voronoi tessellations, we generalize the construction of [1] to the unweighted case. Specifically, we map every domain of ${\mathrm{Vor}_{k}{({X})}}$ to the sum of the corresponding *k* points, for the moment ignoring the weights. For every non-empty common intersection of domains, we collect the images of the domains that contain this intersection, and we add the convex hull of these points as a cell to the dual, which we refer to as the *order-k* *Delaunay mosaic* of *X*, denoted ${\mathrm{Del}_{k}{({X})}}$. Note that this definition is not coordinate invariant, but it can be made so by substituting the average for the sum, which is just a rescaling. We refrain from doing this to simplify the notation.

**Fig. 1:**
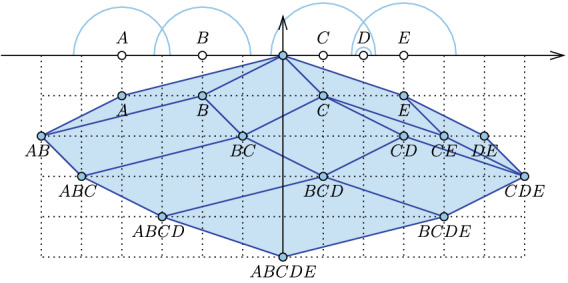
The rhomboid tiling of five weighted points on the real line. The weights are the squared radii of the colored half-circles. The horizontal line at depth *k* intersects the tiling in the order-*k* Delaunay mosaic of the points. Since *D* has much smaller weight than its two neighbors, it is not part of the order-1 mosaic. Nonetheless, *D* participates in all mosaics of order higher than one

We give an alternate description of these mosaics after dualizing the hyperplane arrangement. To this end, we write $y_j = (x_j, -1) \in {{\mathbb {R}}}^{d+1}$, for every $(x_j, w_j) \in X$, and $y_I = \sum _{i \in I} y_i$, for every $I \subseteq J$. The $(d+1)$-st coordinate of $y_I$ is $- {|{I}|}$, and we call $k = {|{I}|}$ the *depth* of the point. For every sphere *S* in ${{\mathbb {R}}}^{d+1}$ — which we recall may be a point or imaginary — we let ${\mathrm{rho}{({S})}} = \mathrm{conv}\,{ \{ y_I \mid {\mathrm{In}{({S})}} \subseteq I \subseteq {\mathrm{In}{({S})}} \cup {\mathrm{On}{({S})}} \} }$ be the *rhomboid* of *S*. It is *anchored* at the vertex with least depth, which is $y_{{\mathrm{In}{({S})}}}$, and it is *spanned* by the vectors $y_i$ with $i \in {\mathrm{On}{({S})}}$. We say *X* is in *general position* if for every collection of $d+2-p \ge 1$ spheres *S* there are at most $p+1$ weighted points that belong to all $d+2-p$ sets ${\mathrm{On}{({S})}}$. For such a set *X*, the dimension of the rhomboid defined by a sphere *S* is the number of vectors that span it, which is $\mathrm{dim}\,{{\mathrm{rho}{({S})}}} = {|{{\mathrm{On}{({S})}}}|}$. he *rhomboid tiling* of *X*, denoted ${\mathrm{Rho}{({X})}}$, is the collection of all rhomboids defined by spheres in ${{\mathbb {R}}}^{d+1}$; see Fig. 1. As argued for the unweighted case in [7], ${\mathrm{Rho}{({X})}}$ is a *polytopal* *complex* in ${{\mathbb {R}}}^{d+1}$, which means its cells are convex polytopes with disjoint interiors such that the boundary of every polytope is the union of other polytopes in the complex [15]. Indeed, we claim the following properties:

### Proposition 2.2

(Rhomboid Tiling) Let *X* be a locally finite set of points with real weights in general position in ${{\mathbb {R}}}^d$. Then

1. ${\mathrm{Rho}{({X})}}$ is dual to ${\mathrm{Arr}{({X})}}$;
2. ${\mathrm{Rho}{({X})}}$ is a polytopal complex of rhomboids;
3. the horizontal slice at integer depth $k \ge 0$ is the order-*k* Delaunay mosaic of *X*.

We refer to the proof of the corresponding result for unweighted points in [7] and note that it readily generalizes to the weighted case. We explain Property 1 of Proposition 2.2 because it is repeatedly used. The duality is established by a bijection ${\rho }\mapsto {\rho }^*$ in which ${\rho }$ is a $(p+1)$-dimensional rhomboid in ${\mathrm{Rho}{({X})}}$ and ${\rho }^*$ is a $(d-p)$-cell in ${\mathrm{Arr}{({X})}}$ such that ${\rho }\subseteq {\varrho }$ iff ${\varrho }^* \subseteq {\rho }^*$. The points inside, on, and outside the sphere that defines ${\rho }$ correspond to the hyperplanes above, containing, under the cell ${\rho }^*$. Note also that the vertical partial order of the cells in the arrangement agrees with the same order on the rhomboids in the tiling.

*Discrete Morse theory* Next we assign to each rhomboid in the tiling a real value. Before doing so, we give a few definitions. Let *K* be a polytopal complex. Its *Hasse diagram* is a directed graph whose nodes are the cells in *K* and whose arcs are the pairs of cells ${\sigma }\subseteq {\tau }$ with $\mathrm{dim}\,{{\tau }} = \mathrm{dim}\,{{\sigma }} + 1$. A function $f :K \rightarrow {{\mathbb {R}}}$ is *monotonic* if $f({\sigma }) \le f({\tau })$ whenever ${\sigma }\subseteq {\tau }$. The *level set* for a value $w \in {{\mathbb {R}}}$ is the set of cells $f^{-1} (w) \subseteq K$. It is a set of nodes in the Hasse diagram. A *step* of *f* is a maximal subset of a level set whose induced subgraph in the Hasse diagram is connected. We note that the steps of *f* partition *K*. An *interval* of *K* is given by cells ${\sigma }\subseteq {\upsilon }$ and consists of all faces of ${\upsilon }$ that share ${\sigma }$ as a face, denoted $[{\sigma }, {\upsilon }] = \{ {\tau }\in K \mid {\sigma }\subseteq {\tau }\subseteq {\upsilon }\}$. We call ${\sigma }$ the *lower bound* and ${\upsilon }$ the *upper bound* of $[{\sigma }, {\upsilon }]$. The interval is *singular* if ${\sigma }= {\upsilon }$. A monotonic function $f :K \rightarrow {{\mathbb {R}}}$ is *generalized discrete Morse* if every step is an interval; see [11]. For comparison, *f* is *discrete Morse* if every step is an interval of size 1 or 2; see [10] but note that the original definition is in-essentially more general by allowing $f({\sigma }) > f({\tau })$ for pairs ${\sigma }\subseteq {\tau }$ in a step.

We are interested in the case in which $K = {\mathrm{Rho}{({X})}}$ and *f* is the squared radius function, which we now define. Recall that each rhomboid ${\rho }\in {\mathrm{Rho}{({X})}}$ has a dual cell ${\rho }^* \in {\mathrm{Arr}{({X})}}$. For each point $y = (x, z) \in {\rho }^*$, we set $w(y) = {\Vert {x}\Vert }^2 - 2 z$, and we define the *squared radius function*, ${\mathbf{w}}:{\mathrm{Rho}{({X})}} \rightarrow {{\mathbb {R}}}$, by mapping ${\rho }$ to ${\mathbf{w}}({\rho }) = \min _{y \in {\rho }^*} w(y)$. To develop a geometric intuition for this function, we sweep the hyperplane arrangement from top to bottom with a paraboloid. Specifically, for $t \in {{\mathbb {R}}}$, the paraboloid is the graph of $\varpi - \tfrac{1}{2} t$. Then ${\mathbf{w}}({\rho })$ is the minimum $t \in {{\mathbb {R}}}$ such that the corresponding paraboloid has a non-empty intersection with ${\rho }^*$. Assuming general position, the sequence in which the paraboloid encounters the cells in ${\mathrm{Arr}{({X})}}$ follows a few simple rules. For example, when the paraboloid encounters a vertex in ${\mathrm{Arr}{({X})}}$, then it has already encountered $2^{d+1} - 1$ of the chambers incident to the vertex, and it touches the unique last incident chamber for the first time, as well as all faces of this chamber that share the vertex. More generally, when the paraboloid touches the intersection of $p+1$ hyperplanes for the first time, this happens at an interior point of a $(d-p)$-cell contained in this intersection. At the same time the paraboloid touches a unique chamber together with all faces of this chamber that share the $(d-p)$-cell as a common face. We therefore have the following result.

### Proposition 2.3

(Squared Radius Function) Let *X* be a locally finite set of points with real weights in general position in ${{\mathbb {R}}}^d$. Then ${\mathbf{w}}:{\mathrm{Rho}{({X})}} \rightarrow {{\mathbb {R}}}$ is a generalized discrete Morse function. Furthermore, every step of ${\mathbf{w}}$ has a vertex as a lower bound, and there is only one singular interval, namely the vertex at the origin, which corresponds to the empty index set.

The same claim restricted to the unweighted case can be found in [7]. Its proof extends with obvious modifications to the proof in the weighted case, which we therefore omit. This proposition says that the paraboloid always enters a chamber together with a subset of its faces while sweeping, thus defining a *shelling* of ${\mathrm{Arr}{({X})}}$ (see [4]). This chamber corresponds to the vertex of a rhomboid and the faces of the chamber correspond to the faces of the rhomboid that share this vertex. As we will see shortly, the vertex is not necessarily the lowest vertex of the rhomboid. Note that ${\mathbf{w}}$ is rather special because it has a limited collection of interval types. This is best seen by constructing ${\mathrm{Rho}{({X})}}$ one step at a time. After starting with the vertex at the origin, each step glues a new rhomboid of dimension at least 1 together with all missing faces to the complex. Such a step preserves the homotopy type, which implies that every non-empty sublevel set of ${\mathbf{w}}$ is contractible.

*Horizontal integer slices* Let ${{P}_{k}}$ be the horizontal hyperplane at integer depth *k* in ${{\mathbb {R}}}^{d+1}$. By Proposition 2.2, the intersection of ${{P}_{k}}$ with ${\mathrm{Rho}{({X})}}$ gives the (weighted) order-*k* Delaunay mosaic. We aim at associating each cell in ${\mathrm{Del}_{k}{({X})}}$ with the rhomboid in ${\mathrm{Rho}{({X})}}$ such that the cell is the intersection of ${{P}_{k}}$ with the rhomboid, but there is an ambiguity for the vertices of ${\mathrm{Del}_{k}{({X})}}$, which belong to several intersections. We will associate them to the vertices of ${\mathrm{Rho}{({X})}}$, but to avoid special cases, we formulate the definitions for the rhomboids without their boundary. Note that the (relative) interiors of the rhomboids partition the union of the rhomboids. Accordingly, each cell ${\sigma }\in {\mathrm{Del}_{k}{({X})}}$ is the closure of the intersection of ${{P}_{k}}$ with the interior of a unique rhomboid ${\rho }= {\rho }({\sigma })$. Writing $p = \mathrm{dim}\,{{\sigma }}$, we have $\mathrm{dim}\,{{\rho }({\sigma })} = p+1$ if $p \ge 1$, and $\mathrm{dim}\,{{\rho }({\sigma })} = 0$ if $p = 0$. Similarly, we get the *squared radius function* of ${\mathrm{Del}_{k}{({X})}}$ from that of ${\mathrm{Rho}{({X})}}$: ${\mathbf{w}_{k}^{}} ({\sigma }) = {\mathbf{w}}({\rho }({\sigma }))$. We are interested in the partition of ${\mathrm{Del}_{k}{({X})}}$ into the steps of ${\mathbf{w}_{k}^{}}$. To this end, let ${\sigma }$ and ${\tau }$ be two cells in ${\mathrm{Del}_{k}{({X})}}$ and note that ${\sigma }\subseteq {\tau }$ iff ${\rho }({\sigma }) \subseteq {\rho }({\tau })$. It follows that each step of ${\mathbf{w}_{k}^{}}$ is the horizontal slice of a step of ${\mathbf{w}}$.

Letting ${\rho }\in {\mathrm{Rho}{({X})}}$, we write ${\mathrm{top}{({{\rho }})}}$ and ${\mathrm{btm}{({{\rho }})}}$ for the vertices with minimum and maximum depth, and we write ${\mathrm{last}{({{\rho }})}}$ for the vertex with maximum value of ${\mathbf{w}}$. Proposition 2.3 implies that $\lambda = {\mathrm{last}{({{\rho }})}}$ for every interval $[\lambda , {\rho }]$ of ${\mathbf{w}}:{\mathrm{Rho}{({X})}} \rightarrow {{\mathbb {R}}}$. For some rhomboids, we have ${\mathrm{last}{({{\rho }})}} = {\mathrm{btm}{({{\rho }})}}$, but not necessarily for all. Depending on the shape of the rhomboid, $\lambda$ can indeed be any vertex of ${\rho }$ other than ${\mathrm{top}{({{\rho }})}}$. We formally state this as a lemma:

### Lemma 2.4

(Last not Top Vertex) Let *X* be a locally finite set of points with real weights in general position in ${{\mathbb {R}}}^d$. Then $\lambda \ne {\mathrm{top}{({{\rho }})}}$ for every non-singular interval $[\lambda , {\rho }]$ of ${\mathbf{w}}$.

Indeed, the chamber in ${\mathrm{Arr}{({X})}}$ that is dual to ${\mathrm{top}{({{\rho }})}}$ lies above ${\rho }^*$. Since $\rho$ is an upper bound, the point at which the paraboloid first touches ${\rho }^*$ during the sweep is an interior point. Hence, ${\mathrm{top}{({{\rho }})}}^*$ has a lower value of ${\mathbf{w}}$ and therefore does not belong to the interval.

## Topology of a step

This section proves our main result: that critical and non-critical steps of ${\mathbf{w}_{k}^{}}$ can be distinguished by whether ${\mathrm{last}{({{\rho }})}}$ is equal to or different from ${\mathrm{btm}{({{\rho }})}}$, with ${\rho }$ the smallest rhomboid in ${\mathrm{Rho}{({X})}}$ that contains the corresponding step of ${\mathbf{w}}$. The addition of a critical step changes the homotopy type of the sublevel set and the addition of a non-critical step preserves it. We begin with an enumeration of the types.

**Fig. 2:**
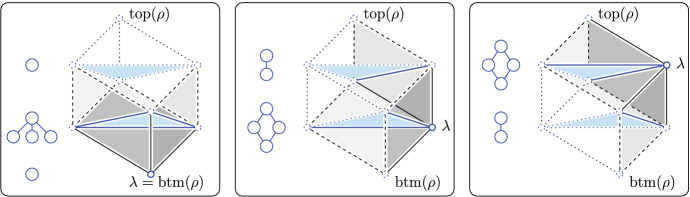
A 3-rhomboid ${\rho }$ with dashed silhouette separating the (gray) faces that share $\lambda$ from the (transparent) other faces. Each slice is blue and shown together with the corresponding subgraph of the Hasse diagram of the Delaunay mosaic of the corresponding order. Left: the self-centered configurations whose corresponding critical steps consist of a triangle without its boundary, a triangle with its edges but without vertices, and a vertex. Middle and right: the altruistic configurations whose corresponding non-critical steps consist in both cases of a triangle with one edge, and a triangle with two edges and the shared vertex

*Step types* By Proposition 2.3, every step of ${\mathbf{w}}:{\mathrm{Rho}{({X})}} \rightarrow {{\mathbb {R}}}$ is an interval $[\lambda , {\rho }]$ in which ${\rho }$ is the maximum rhomboid that satisfies $\lambda = {\mathrm{last}{({{\rho }})}}$. The interval consists of all faces of ${\rho }$ that share $\lambda$. Assuming $\mathrm{dim}\,{{\rho }} = p + 1 \ge 0$, the rhomboid has vertices at $p+2$ depth values, and letting *k* be the depth value of ${\mathrm{btm}{({{\rho }})}}$, these values are $k-g$ for $0 \le g \le p+1$. By Lemma 2.4, $\lambda$ can assume only $p+1$ of these depth values. If $\lambda \ne {\mathrm{btm}{({{\rho }})}}$, then $P_{k-g}$ has a non-empty intersection with the interior of at least one rhomboid in the interval for $1 \le g \le p$, and if $\lambda = {\mathrm{btm}{({{\rho }})}}$, then there is one more, namely for $0 \le g \le p$. In total, we count $p^2 + p + 1$ possible types of slices; see Fig. 2 for an illustration of the types for $p=2$. Some of these types are symmetric. We refer to the $p+1$ slices in case $\lambda = {\mathrm{btm}{({{\rho }})}}$ as *self-centered* and the $p^2$ other slices as *altruistic*. The terminology is motivated by the fact that ${\mathrm{last}{({{\rho }})}} = {\mathrm{btm}{({{\rho }})}}$ iff the convex hull of the points whose indices are in ${\mathrm{On}{({S})}}$ contain the center of *S*. There is an ambivalent case, when the center lies on the boundary of the convex hull, but this can be prevented by slightly strengthening the general position assumption to forbid this situation.

*Topology type* Letting $A \subseteq {\mathrm{Rho}{({X})}}$ be a step of ${\mathbf{w}}$, we write ${{P}_{k}} \cap A \subseteq {\mathrm{Del}_{k}{({X})}}$ for the corresponding step of ${\mathbf{w}_{k}^{}}$. The *Euler characteristic* of *A* is ${\chi }{({A})} = \sum _{{\rho }\in A} (-1)^{\mathrm{dim}\,{{\rho }}}$. Since *A* is necessarily an interval, its Euler characteristic vanishes, unless $A = \{0\}$, in which case it is 1. The Euler characteristic of the slice is ${\chi }{({{{P}_{k}} \cap A})} = \sum _{{\tau }\in {{P}_{k}} \cap A} (-1)^{\mathrm{dim}\,{{\tau }}}$, which may or may not be zero. We write |*A*| for the union of interiors of the rhomboids in *A*, and ${{P}_{k}} \cap |A|$ for its slice at depth *k*. Let ${{\mathbb {H}}}^p \subseteq {{\mathbb {R}}}^p$ be the set of points with non-negative first coordinate, and note that ${\chi }{({{{\mathbb {H}}}^p})} = 0$ for all $p \ge 1$. Two topological spaces have the same *topology type* if there is a homeomorphism between them, and in this case they have the same Euler characteristic. For example, the half-open interval, [0, 1), has the same topology type as ${{\mathbb {H}}}^1$, which we denote as $[0,1) \approx {{\mathbb {H}}}^1$. We represent [0, 1) by an edge together with one of its endpoints, so the Euler characteristic, which is the alternating sum of cells vanishes. We will see that every altruistic configuration has the topology type of ${{\mathbb {H}}}^p$, for some value of *p*, while every self-centered configuration has non-zero Euler characteristic.

### Theorem 3.1

(Topology of a Step) Let *X* be a locally finite set of points with real weights in general position in ${{\mathbb {R}}}^d$, let $A = [\lambda , {\rho }]$ be a step of ${\mathbf{w}}$, set $p+1 = \mathrm{dim}\,{{\rho }}$, write *k* for the depth of ${\mathrm{btm}{({{\rho }})}}$, and recall that ${{P}_{k-g}} \cap A$ is a step of ${\mathbf{w}_{k-g}^{}}$.

1. If ${\mathrm{last}{({{\rho }})}} = {\mathrm{btm}{({{\rho }})}}$, then ${\chi }{({{{P}_{k-g}} \cap A})} \ne 0$ for $0 \le g \le p$.
2. If ${\mathrm{last}{({{\rho }})}} \ne {\mathrm{btm}{({{\rho }})}}$, then ${{P}_{k-g}} \cap |A| \approx {{\mathbb {H}}}^p$ and therefore ${\chi }{({{{P}_{k-g}} \cap A})} = 0$ for $1 \le g \le p$.

All other horizontal integer slices of *A* are empty.

**Fig. 3:**
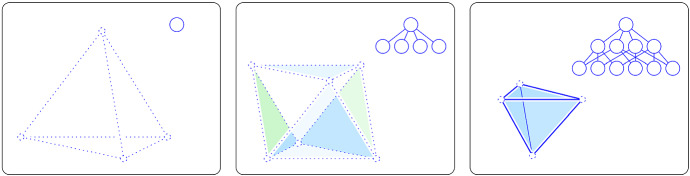
Three self-centered configurations in ${{\mathbb {R}}}^3$. From left to right: a tetrahedron without boundary faces, an octahedron with four of its triangles but no other faces, and a tetrahedron with all of its faces except for the vertices

**Proof **We first consider the self-centered configurations, when $\lambda = {\mathrm{last}{({{\rho }})}} = {\mathrm{btm}{({{\rho }})}}$. For $g = 0$, the hyperplane ${{P}_{k-g}}$ contains $\lambda$ and avoids the interiors of all other rhomboids in $A = [\lambda , {\rho }]$. The Euler characteristic of this slice is one and therefore non-zero, as claimed. For $1 \le g \le p$, ${{P}_{k-g}}$ has non-empty intersections with the interiors of the rhomboids of dimension larger than *g* and empty intersections with the interiors of all other rhomboids in the interval; see the left panel of Fig. 2 for the three cases that occur for $p=2$, and see Fig. 3 for three of the four cases that occur for $p=3$. Therefore, $${\chi }{({{{P}_{k-g}} \cap A})} = \sum _{q=g+1}^{p+1} (-1)^{q} \left( {\begin{array}{c}p+1\\ q\end{array}}\right) $$, in which the binomial coefficient is the number of *q*-dimensional faces of a $(p+1)$-dimensional rhomboid that share a common vertex, namely $\lambda$. This sum evaluates to $$(-1)^{g+1} \left( {\begin{array}{c}p\\ g\end{array}}\right) $$, which is non-zero, as claimed.

We second consider the altruistic configurations, when $\lambda \ne {\mathrm{btm}{({{\rho }})}}$. Let $\lambda '$ be the vertex of ${\rho }$ opposite to $\lambda$, and project ${\rho }$ orthogonally to the hyperplane normal to $\lambda ' - \lambda$. The projection is a *p*-dimensional convex polytope. Call the preimage of its (relative) boundary the *silhouette* of ${\rho }$, and note that it is a $(p-1)$-dimensional topological sphere that contains all vertices of ${\rho }$ other than $\lambda$ and $\lambda '$; see Fig. 2. None of the rhomboids in the silhouette belong to $A = [\lambda , {\rho }]$. In fact, the silhouette separates the boundary rhomboids of $\rho$ that are in this interval from the boundary rhomboids that are not in the interval. Since ${\mathrm{btm}{({{\rho }})}}$ and ${\mathrm{top}{({{\rho }})}}$ belong to the silhouette, ${{P}_{k}}$ and ${{P}_{k-(p+1)}}$ both have empty *intersection* with the interiors of all rhomboids in $[\lambda , {\rho }]$, as claimed.

**Fig. 4:**
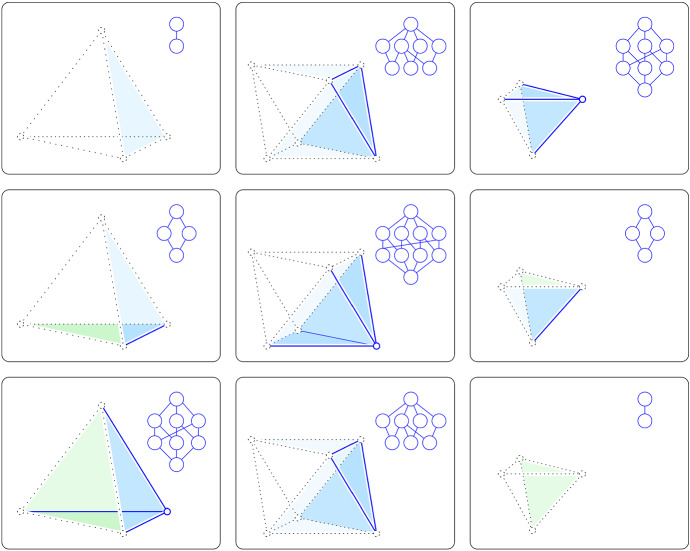
The 9 altruistic configurations in ${{\mathbb {R}}}^3$. Compared to ${\mathrm{btm}{({{\rho }})}}$ at depth *k*, the vertex $\lambda = {\mathrm{last}{({{\rho }})}}$ has depth $k-3$, $k-2$, and $k-1$ in the *top*, *middle*, and *bottom row*. Similarly, the slice is at depth $k-3$, $k-2$, and $k-1$ in the left, middle, and right column

We thus assume $1 \le g \le p$ for the remainder of this proof. At depth $k-g$, the horizontal hyperplane intersects ${\rho }$ in a convex polytope of dimension *p*, and it intersects the boundary of ${\rho }$ on both sides of the silhouette. To go from one side to the other along the boundary of ${\rho }$ intersected with ${{P}_{k-g}}$, we have to cross the intersection of ${{P}_{k-g}}$ with the silhouette, which we will prove is a topological $(p-2)$-sphere. We conclude that an open $(p-1)$-ball of the boundary belongs to ${{P}_{k-g}} \cap |A|$, and the complementary closed $(p-1)$-ball does not belong to ${{P}_{k-g}} \cap |A|$. It follows that the slice of the interval has the topology type of ${{\mathbb {H}}}^p$, as claimed. The middle and right panels of Fig. 2 illustrate the four altruistic configurations for $p=2$, and Fig. 4 illustrates the nine altruistic configurations for $p=3$. Reading the eight outer cases in a circle around the center case, we note that each is symmetric to the diagonally opposite type. In other words, there are really only five altruistic types for $p = 3$.

We return to the intersection of ${{P}_{k-g}}$ with the silhouette and the claim that this intersection is a topological sphere of dimension $p-2$. For $p=1$, ${\rho }$ is a convex quadrangle, its silhouette consists of two vertices, ${\mathrm{top}{({{\rho }})}}$ and ${\mathrm{btm}{({{\rho }})}}$, and ${{P}_{k-1}}$ passes through the other two vertices thus intersecting the silhouette in the empty set—the $(-1)$-sphere—as claimed. Assuming $p \ge 2$, we denote the silhouette by ${{\mathbb {S}}}$, we recall that it is a $(p-1)$-sphere, and we write $e :{{\mathbb {S}}}\rightarrow {{\mathbb {R}}}$ for the depth function on the silhouette. Its extreme values are $e( {\mathrm{top}{({{\rho }})}} ) = k-(p+1)$ and $e( {\mathrm{btm}{({{\rho }})}} ) = k$, and ${{P}_{k-g}} \cap {{\mathbb {S}}}= e^{-1} (k-g)$. To prove that this level set is a $(p-2)$-sphere, it suffices to show that *e* has only two critical points, namely the minimum at ${\mathrm{top}{({{\rho }})}}$ and the maximum at ${\mathrm{btm}{({{\rho }})}}$. The case $p=2$ is easy. Here we have a 3-rhomboid whose silhouette is a hexagon. The difference between the depths of the endpoints of any edge is 1. We thus need three edges to go from ${\mathrm{btm}{({{\rho }})}}$ at depth *k* to ${\mathrm{top}{({{\rho }})}}$ at depth $k-3$ and another three edges to go back. It follows that ${{P}_{k-g}}$ meets the silhouette in two points—a 0-sphere—as claimed.

The argument for $p>2$ is different. Recall that *e* is a continuous function on a $(p-1)$-sphere, this sphere is decomposed into $(p-1)$-rhomboids, and *e* is affine on each of these rhomboids. If *e* has a critical point (in piecewise-linear sense) in addition to the minimum at ${\mathrm{top}{({{\rho }})}}$ and the maximum at ${\mathrm{btm}{({{\rho }})}}$, then it also has a saddle, and this saddle must be a vertex of some of the rhomboids. To contradict the existence of a saddle, note that the $(p-1)$-rhomboids meet in groups of *p* at a common vertex. Let $\nu$ be such a shared vertex and cut each incident $(p-1)$-rhomboid with the $(p-2)$-dimensional plane that passes through the vertices adjacent to $\nu$. We thus get *p* $(p-1)$-simplices, which can be seen are the facets of a *p*-simplex. It follows that $\nu$ can be a minimum, a maximum, or a regular point, but it cannot be a saddle of *e*. Hence, every horizontal slice at depth strictly between $k-(p+1)$ and *k* is a $(p-2)$-sphere, as required. $\square$

*Consequences* Our main technical result is Theorem 3.1, which we now turn into a statement about the filtration of order-*k* Delaunay mosaics. Let $w_0< w_1 < \ldots$ be the sorted values of ${\mathbf{w}_{k}^{}}$ and write $K_\ell = {\mathbf{w}_{k}^{-1}} [-\infty , w_\ell ] \subseteq {\mathrm{Del}_{k}{({X})}}$ for every $\ell \ge 0$. Assuming *X* is in general position, the difference between any two contiguous mosaics is a collection of steps, and by slightly strengthening the notion of general position, we may assume that each difference is a *single* step: $A_\ell = K_\ell \setminus K_{\ell -1}$. For example, all vertices of the order-1 Delaunay mosaic of unweighted points share the function value, 0, and we can perturb the set by assigning small weights. While it is not necessary, we simplify the following statement by using this stronger notion of general position.

### Corollary 3.2

(Filtration of Order-k Delaunay Mosaics) Let *X* be a locally finite set of points with real weights in general position in ${{\mathbb {R}}}^d$, and let $0 \le k$ and $0 \le u \le v$ be integers.

1. If exactly one of the steps $A_u, A_{u+1}, \ldots , A_v$ of ${\mathbf{w}_{k}^{}}$ is critical, then $K_u$ and $K_v$ have different Euler characteristics and therefore different homotopy types.
2. If $A_u, A_{u+1}, \ldots , A_v$ are all non-critical steps of ${\mathbf{w}_{k}^{}}$, then $K_u$ and $K_v$ have the same homotopy type.

This corollary of Theorem 3.1 is a direct extension of a theorem about discrete Morse functions in [10]. Other results in this theory can be similarly extended.

## Discussion

The main result of this paper is a topological characterization of the incremental steps of the squared radius function on the order-*k* Delaunay mosaic of a locally finite set of possibly weighted points in Euclidean space. With this insight, we gain a topological interpretation of the probabilistic analysis of this function for a stationary Poisson point process [6]. While the critical steps do not determine the topology of the sublevel sets, they provide bounds on the ranks of their homology groups. In contrast to the order-1 case studied in [2], the squared radius function in the order-*k* case is neither discrete Morse nor generalized discrete Morse [10, 11]. Since the function nevertheless behaves similar to a Morse function, it may be considered a geometrically motivated further extension of the framework; see also [12, Chapter 11] for algebraically motivated extensions of discrete Morse theory.

In conclusion, we mention that our result requires the given points be in general position. While this assumption does not imply that the Delaunay mosaics are simplicial, it simplifies the analysis by guaranteeing that the dual of the corresponding hyperplane arrangement is a complex of rhomboids. It would be interesting to generalize the theory to locally finite sets that are not necessarily in general position.
